# Advantage of whole-mount histopathology in prostate cancer: current applications and future prospects

**DOI:** 10.1186/s12885-024-12071-6

**Published:** 2024-04-11

**Authors:** Lewei Duan, Zheng Liu, Fangning Wan, Bo Dai

**Affiliations:** 1 Department of Urology, Fudan University Shanghai Cancer Center, 200032 Shanghai, China; 2 Department of Oncology, Shanghai Medical College, Fudan University, 200032 Shanghai, China; 3 Shanghai Genitourinary Cancer Institute, 200032 Shanghai, China

**Keywords:** Whole-mount histopathology, Prostate cancer, MRI, AI, Histopathology

## Abstract

**Background:**

Whole-mount histopathology (WMH) has been a powerful tool to investigate the characteristics of prostate cancer. However, the latest advancement of WMH was yet under summarization. In this review, we offer a comprehensive exposition of current research utilizing WMH in diagnosing and treating prostate cancer (PCa), and summarize the clinical advantages of WMH and outlines potential on future prospects.

**Methods:**

An extensive PubMed search was conducted until February 26, 2023, with the search term “prostate”, “whole-mount”, “large format histology”, which was limited to the last 4 years. Publications included were restricted to those in English. Other papers were also cited to contribute a better understanding.

**Results:**

WMH exhibits an enhanced legibility for pathologists, which improved the efficacy of pathologic examination and provide educational value. It simplifies the histopathological registration with medical images, which serves as a convincing reference standard for imaging indicator investigation and medical image-based artificial intelligence (AI). Additionally, WMH provides comprehensive histopathological information for tumor volume estimation, post-treatment evaluation, and provides direct pathological data for AI readers. It also offers complete spatial context for the location estimation of both intraprostatic and extraprostatic cancerous region.

**Conclusions:**

WMH provides unique benefits in several aspects of clinical diagnosis and treatment of PCa. The utilization of WMH technique facilitates the development and refinement of various clinical technologies. We believe that WMH will play an important role in future clinical applications.

## Background

Whole-mount histopathology (WMH) is a histopathologic technique that enables the examination of the intact cross-section of prostatic specimen, which is not feasible with conventional histopathologic methods. WMH has been admitted as a useful approach that was recommended to be adopted in routine urologic examination [1]. It provides a more comprehensive understanding of the morphologic feature and spatial distribution of prostate cancer (PCa) lesions compared to conventional histopathologic methods, which can be critical for tumor classification and the selection of appropriate treatment strategies. It also provides a more accurate assessment of tumor extension, thereby providing important insights into the progression and prognosis of prostate cancer.

**Fig. 1:**
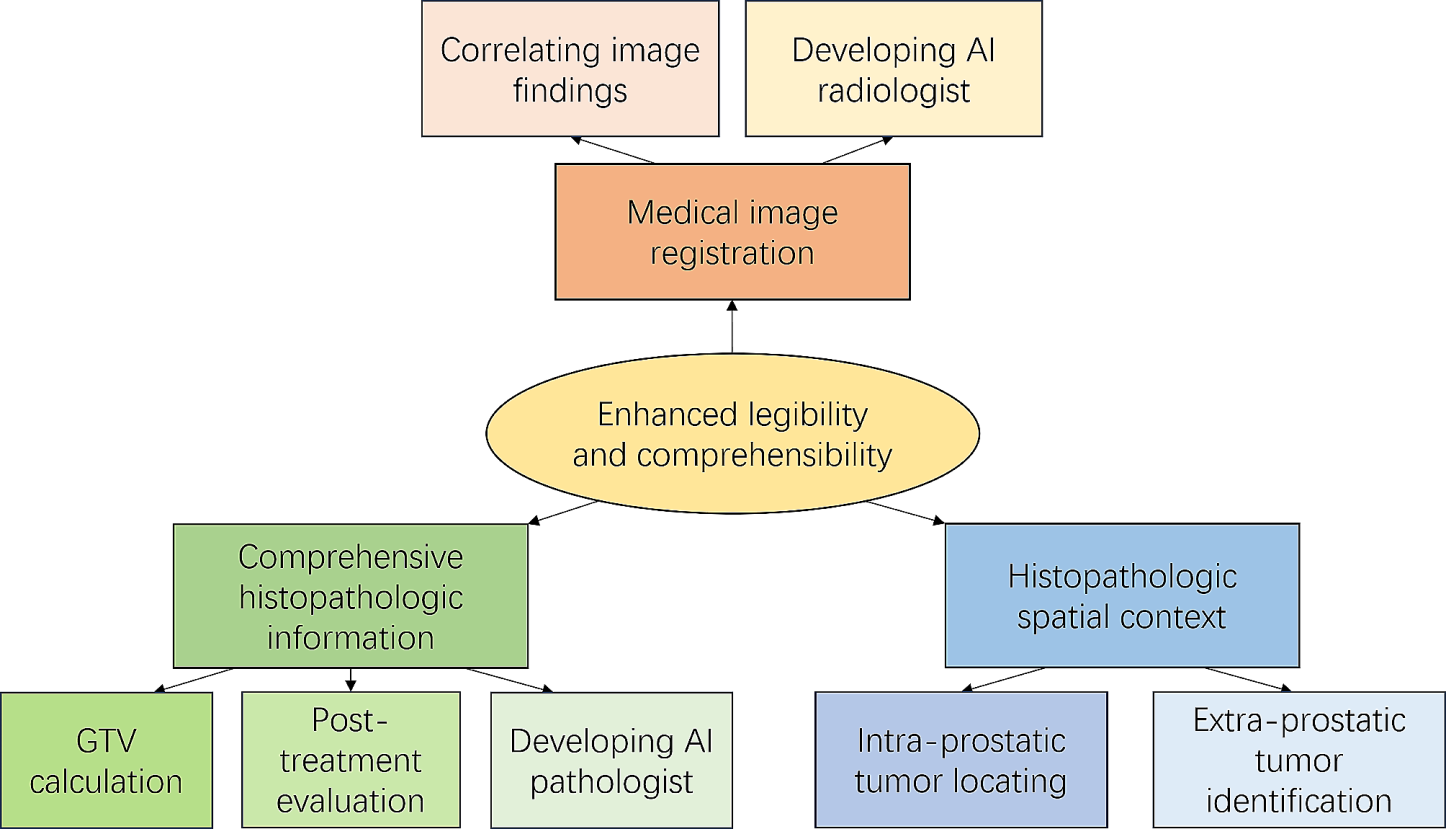
The application of Whole Mount Histopathology in clinical diagnosis and treatment of prostate cancer. AI = artificial intelligence; GTV = gross tumor value

Despite the existence of several reviews elucidating the clinical value of WMH [1, 2], recent advancement in diagnosis and treatment technologies significantly expanded its applications, especially in the development of AI methodology, which has not yet been summarized. Hence, the objective of this article is to provide a comprehensive overview of the advantage of WMH comparing to conventional histopathology, and summary its significance in current clinical application for the diagnosis and treatment of PCa. Finally, we discussed and analyzed the potential value of WMH technique and proposed its future prospects **(**Fig. 1a**)**.

## Methods

Our search was performed on PubMed on February 26, 2023, using the query “prostate[tiab] AND (“whole-mount“[tiab] OR “large format histology“[tiab])”. The search was restricted to English-published articles. To limit the number of citations and ensure the currency of included articles in our review, we only included articles published after 2019. We include only research articles in article type screening, with review articles, editorials, and comments etc. excluded. Titles and abstracts were utilized to screen for research goal and WMH methodology, with full texts reviewed when abstract lacked sufficient information. For the research goal, the research included should be of assistance or potential assistance in the clinical diagnosis and treatment of prostate cancer. As for WMH methodology, we restricted the study material to HE-stained human prostate specimens. Other papers are cited if they contributed to a better understanding.

## Results

**Fig. 2:**
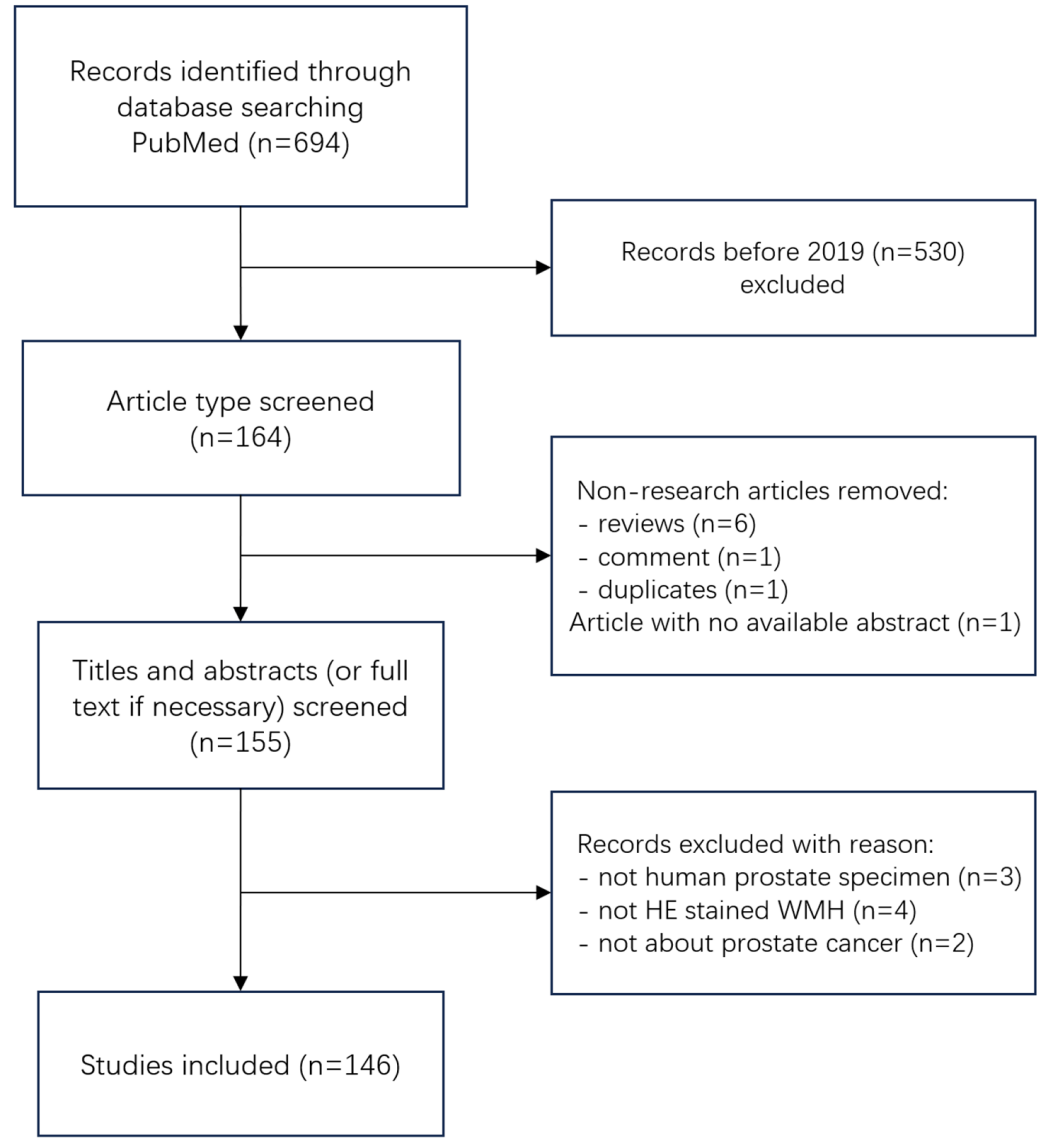
Flowchart of article acquisition and selection process. WMH = whole mount histopathology

As depicted in Fig. 2, the search yielded a total of 694 articles, which were subsequently subjected to evaluation. In publication time screening, 530 articles were excluded for their publication was before 2019. We then proceeded article type screening to the remaining 164 records, in which six reviews, one comment and one duplicate article were excluded, and one article was removed for having no available abstract. In the title and abstract screening for research goal and WMH methodology, nine articles were excluded, in which two were not studying prostate cancer, mismatching the inclusion criteria for research goal; three were using non-human prostate, and four were using non-HE-stained specimen, mismatching the inclusion criteria of WMH methodology. Finally, we included 146 articles in our review study.

### WMH section demonstrates enhanced legibility for pathologist

Whole-mount histopathology (WMH) maintain the integrity of tissue section during embedding and sectioning. The application of WMH presents precise information that is not only helpful in prostate cancer (PCa) diagnosis and prognosis, but also have clinical significance or potential clinical significance [2].

#### General legibility

**Fig. 3:**
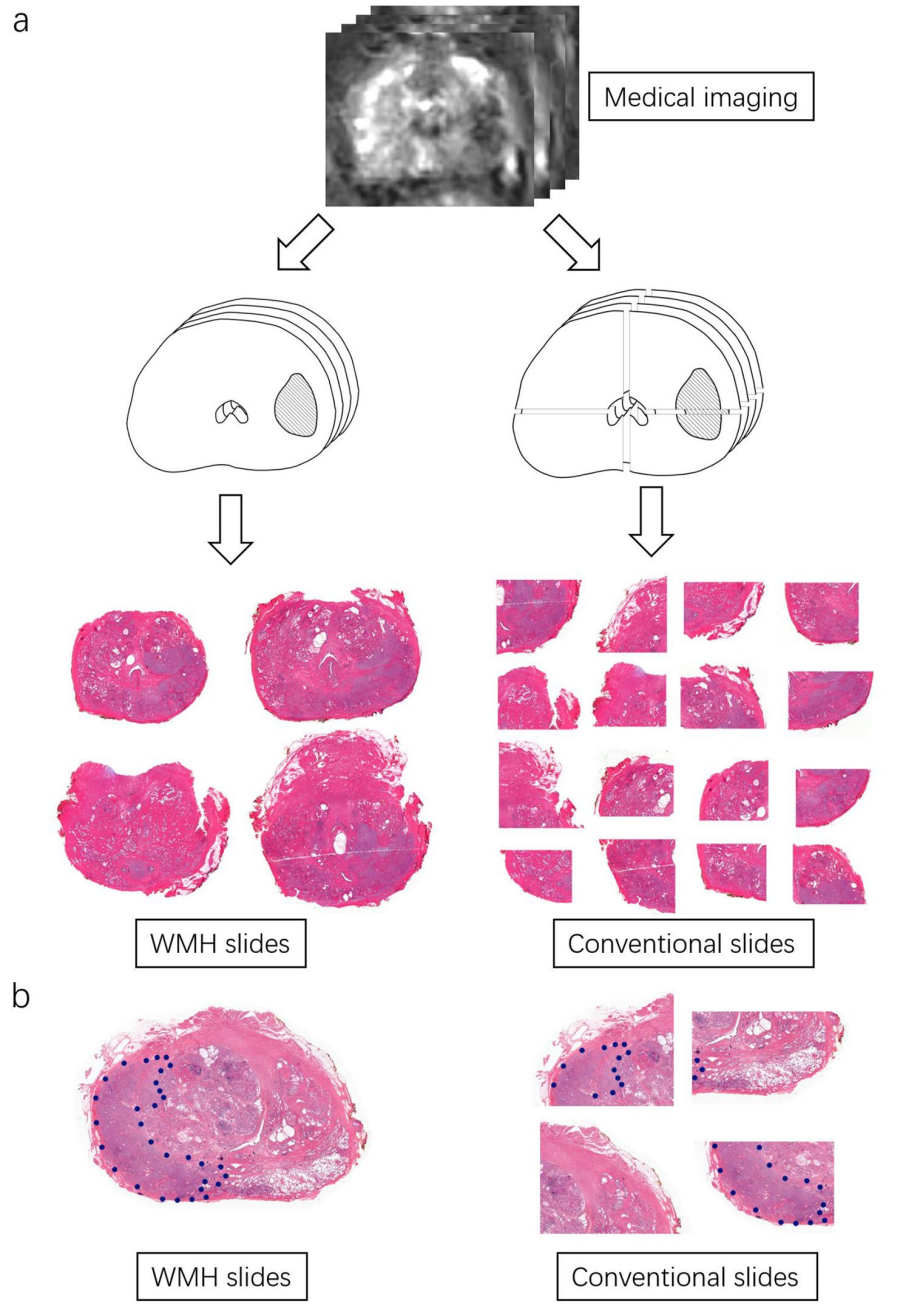
WMH demonstrates enhanced legibility for pathologist. **(a) WMH demonstrates enhanced general legibility.** It is hard to recognize an intact cross-section image in conventional histopathologic sections. In contrast, WMH preserves the intact cross-section and provides more comprehensive view of intact cross-section. **(b) WMH demonstrates enhanced lesion-scale legibility**. The disordered arrangement complicates the examination of large tumor lesion, and the cutting edges may cause missing or distortion of the prostatic tissue. While WMH provides a complete and comprehensible view of the large tumor lesion. WMH = whole mount histopathology

In conventional histopathology sampling, each tissue slice was divided into at least four quadrants before embedding. Due to the flipping and rotating of prostatic tissue on the histopathological sections, it becomes challenging to recognize a complete cross-section structure from multiple sections. In contrast, WMH preserved the intact cross-section surface of the prostate, providing a better correspondence to its anatomical structure and offering pathologists the convenience of obtaining a comprehensive view of the intact cross-section surface **(**Fig. 3a**)**, which would be more acceptable to pathologist, and helpful for them to identify deviations from the normal features [2]. The visual legibility of WMH also contributes significantly to the enhancement of clinical understanding among pathologists and radiologists [2].

#### Lesion-scale legibility

Conventional histopathology is not well compatible with large tumor lesion that exceed the size of paraffin slides. The disordered arrangement of prostatic tissue on the histopathologic sections posts challenges in identifying identical tumors from multiple sections. In comparison, WMH preserve the structural integrity of intraprostatic tumor lesions and consistently display them on a single slide, especially for large tumors or multiple lesions. In recent nerve sparing surgery or focal therapy, the location of tumor matters a lot. WMH will provide more evidence for these patients and urologist for decision making and procedure planning. **(**Fig. 3b**)**.

The enhanced legibility WMH demonstrated improves the efficiency of histopathologic examination. WMH demonstrated higher detection rate of adverse pathological events [3] (e.g. positive surgical margin) and yielded new information in 2.1% of patients [4], comparing to conventional histopathology. Considering that whole-mount sampling reduces the variability between different operators, it can improve the consistency and reliability of pathologic examination. Moreover, the well-preserved structural integrity provided by WMH sections can serve as the basis for histopathologic evaluation, thereby considerably reducing the challenges and improve the efficacy of the identification of significant pathological features.

The enhanced legibility of WMH also provides irreplaceable educational benefits for personnel training. For the inexperienced pathologists, because the appearance of WMH section is the same as its anatomy status, the challenges for identifying pathologic features has been largely reduced, which facilitates the understanding and learning process for pathologists. What’s more, the legibility of WMH facilitates its registration with medical imaging (which will be elaborated in Sect. 3.2), in which inexperienced radiologist can learn from the relationship between correlated imaging features and pathological features, therefore benefiting the education of radiologist.

### WMH simplify histopathological registration with medical images

Histopathologic registration is a procedure that aligns histopathologic images with corresponding medical images obtained from the same tissue sample [5]. WMH preserves the complete structure of prostatic cross-section, thus provides direct comparison between medical imaging findings and histopathologic features. Moreover, WMH exhibited higher detection rate of adverse pathological events, thus improves the reliability of histopathologic reference standard in comparison with conventional histopathology. These advantages brought by WMH can facilitate the development and enhancement of medical imaging methods.

#### WMH facilitate correlation between imaging findings and histopathologic features

Two methods can be employed to identify correlations between imaging findings and histopathologic features. For the sector-based registration, the prostate is divided into small sectors, where suspected lesions and cancerous changes were calculated on a sector basis. For the complete spatial registration, a patient-specific mold is needed to ensure the orientation and interval of WMH slices are the same as medical images, enabling their complete spatial overlap. It demonstrated superior accuracy and repeatability than sector-based registration, but requires special materials and machines, thus have more limitations in implementation.

1. *Magnetic Resonance Imaging*: By registering with WMH, some features on Magnetic Resonance images exhibit discernible association with PCa characteristic and distribution, therefore can be utilized in PCa detection and classification. Studies have demonstrated the efficacy of Prostate Imaging Reporting and Data System (PI-RADS) in PCa diagnosis with WMH registration, with most achieving significant accuracy [6–8]. There are also correlations between WMH-obtained tumor feature and specific indicator in Magnetic Resonance Imaging (MRI) sequences. Lower apparent diffusion coefficient (ADC) value exhibits a correlation with higher cancerous degree, thus indicating the ability to identify tissue malignancy [9–11]. For dynamic contrast enhanced (DCE) images, malignant region correlates with early enhancement [10], and time-to-peak (TTP) outperformed ADC in low-grade tumor detection [9]. There are also conflicts between studies about the performance of ADC in different regions that needs further considerations [11, 12].

The development of new MRI techniques is also reliant to WMH registration, as it provides a histopathologic ground truth for evaluating efficacy of MRI methods. In sodium MRI, which can demonstrate the metabolic state of prostatic tissue, the alteration of tissue sodium concentration (TSC) was monotonically correlated to Gleason score [13]. Studies also discovered differentiation between cancerous and benign tissue in hybrid multidimensional (HM) MRI [14] and diffusion-relaxation correlation spectrum imaging (DR-CSI) [15], which can depict the microstructure of prostate tissue. These newly developed MRI techniques possess the potential to reduce the bias of the existing MRI sequences, which lead to the improvement of MRI protocols and imaging guidelines for clinical prostate examination. Detailed information of the above studies is listed in Table 1.

**Table 1:** Studies correlated imaging findings and histopathological features utilizing WMH as the gold standard

Reference	Methods	Indicators	Patients	Methodology	Findings
For MRI studies					
Ageeli 2021 [6]	3T-MRI	PI-RADS	196	prospective single-center	There was a statistically significant correlation between Gleason score and PI-RADS score (χ2 (2, *N* = 196) = 12.838, *p* = 0.002). High PI-RADS on MRI detected more than 80% high risk prostate cancer disease.
Wibulpolprasert 2019 [7]	3T-MRI	PI-RADS	415	retrospective single-center	Detection percentage: (i) index lesions with size ≥ 1 cm: 81.6%; (ii) index lesions with GS ≥ 7: 80.9%; (iii) index lesions with both GS ≥ 7 and size ≥ 1 cm: 83.3%.
Schaudinn 2019 [8]	3T-MRI	PI-RADS	40	retrospective single-center	PI-RADS v2 showed a trend towards lower sensitivities, but differences were not significant for both readers: R1 72.4% (v1) vs. 63.8% (v2) (*P* = 0.426) and R2 77.6% (v1) vs. 69.0% (v2) (*P* = 0.402).
Reynolds 2022 [9]	3T-MRI	ADC, DCE	61	retrospective single-center	The feature importance of ADC was the highest overall, particularly in the high grade tumors. Cohen’s d values or MRI parameters: (i) low grade tumors ADC vs. TTP: 0.323 vs. 0.478; (ii) high grade tumors ADC vs. TTP: 0.959 vs. 0.644.
Chatterjee 2019 [10]	3T-MRI	ADC, DCE	76	retrospective single-center	Most malignant wedge-shaped regions were highly hypointense (10/12; 83%) on ADC maps and showed early enhancement on DCE (7/12; 58%). Mean quantitative ADC value of malignant wedge-shaped regions: 1.13 ± 0.11 μm/ms.
Bajgiran 2019 [11]	3T-MRI	ADC	218	retrospective single-center	The ADC ratio performed better in discriminating PCa lesions with GS = 3 + 3 from those with GS ≥ 3 + 4 comparing to ADC. The AUC value: (i) ADC_tumor_mean_ vs. ADC_ratio_mean_: 0.70 vs. 0.80; (ii) ADC_ratio_min_ vs. ADC_tumor_min_: 0.72 vs. 0.67; *p* = 0.043).
Broeke 2019 [13]	3T Sodium MRI	ΔTSC	10	retrospective single-center	Only the correlation between ΔTSC and Gleason score was statistically significant (rs = 0.791, *p* < 0.01), whereas ADC (rs = -0.306, *p* = 0.079) and ΔT2 (rs = -0.069, *p* = 0.699) were not.
Chatterjee 2022 [14]	hybrid multidimensional (HM) MRI	-	25	prospective single-center	Prostate tissue composition measured with HM MRI and quantitative histologic evaluation did not differ (stroma, 45% ± 11 vs. 44% ± 11, *P* = 0.23; epithelium, 31% ± 15 vs. 34% ± 15, *P* = 0.08; and lumen, 24% ± 13 vs. 22% ± 11, *P* = 0.80).
Zhang 2020 [15]	diffusion-relaxation correlation spectrum imaging (DR-CSI)	fA, fC, fepithelium, flumen	9	prospective single-center	PCa vs. benign tissues: fA: 0.37 ± 0.05 vs. 0.27 ± 0.06; *P* < 0.001; fC: 0.18 ± 0.06 vs. 0.31 ± 0.13; *P* = 0.01); fepithelium: 0.44 ± 0.13 vs. 0.26 ± 0.16; *P* < 0.001: flumen: 0.14 ± 0.08 vs. 0.27 ± 0.18; *P* = 0.004.
For PET studies					
Sonni 2022 [16]	^68^Ga-PSMA-11 PET/CT; 3T-MRI	-	74	prospective single-center	Cancer detection rate (lesion-based): (i) PSMA PET/CT: 85%; (ii) mpMRI: 83%; (iii) PET/CT + mpMRI: 87%. The change in AUC was statistically significant between PSMA PET/CT + mpMRI and the 2 imaging modalities alone for delineation of tumor localization (segment-based analysis) (*P* < 0.001) but not between PSMA PET/CT and mpMRI (*P* = 0.093).
Bahler 2020 [17]	^68^Ga-PSMA-11 PET/CT; 3T-MRI	-	15	prospective single-center	PSMA-positron emission tomography detected 100% of primary/index lesions and 8 of 11 (82%) secondary lesions. All Grade Group 3–5 lesions were detected vs. 12 of 15 Grade Group 2 lesions.
Scheltema 2019 [18]	^68^Ga-PSMA-11 PET/CT; 3T-MRI	-	56	retrospective single-center	PSMA-PET vs. PI-RADS: (i) AUC: 0.91 vs. 0.79; (ii) sensitivity 88% vs. 68%; (iii) specificity 93% vs. 91%.
Gao 2019 [19]	^68^Ga-PSMA-11 PET/CT; 3T-MRI	-	49	retrospective single-center	SUV_max_ differs in cribriform vs. non-cribriform leasions (18.2 vs. 7.2 per lesion, *P* < 0.001) while ADC does not. SUV_max_ can predict cribriform morphology in PCa (odds ratio 11.93, 95% confidence interval 6.49–33.74, per lesion, *P* < 0.001)
Touijer 2019 [20]	^68^Ga-RM2 PET/CT; 3T-MRI	-	16	prospective single-center	The sensitivity, specificity, and accuracy of 68Ga-RM2 PET/CT imaging and mpMRI did not differ significantly. AUC: (i) PET visual analysis: 0.76; (ii) PET quantitative analysis: 0.72; (iii) mpMRI: 0.76; (iv) PET/CT + mpMRI: 0.85.
Alfano 2020 [21]	^18^F-DCFPyL PSMA-PET/MRI	-	12	prospective single-center	A threshold of 67% SUV max with an 8.4 mm margin achieved a sensitivity of 95.0 ± 7.8% and specificity of 76.4 ± 14.7%. A threshold of 81% SUV max with a 5.1 mm margin achieved sensitivity of 65.1 ± 28.4% and specificity of 95.1 ± 5.2%.

ADC = apparent diffusion coefficient; AUC = area under the curve; CT = computed tomography; DCE = dynamic contrast enhanced; EPE = extraprostatic extension; GS = Gleason grade; mpMRI = multiparametric magnetic resonance imaging; MRI = magnetic resonance imaging; PCa = prostate cancer; PET = positron emission tomography; PI-RADS = Prostate Imaging Reporting and Data System; PSMA = prostate-specific membrane antigen; SUV: standardized uptake value; T = Tesla; TSC = tissue sodium concentration; TTP = time-to-peak.

2. *Positron emission tomography*: Co-registration of Positron emission tomography (PET) image and WMH is essential to provide verification standard for PET tracers. With the WMH serving as the ground truth, studies have investigated the efficacy of ^68^Ga-PSMA [16–19], ^68^Ga-RM2 [20] and ^18^F-DCFPyL PSMA [21] in PCa diagnosis. Since the co-registered WMH images can provide a precise histopathologic standard for PET verification, the utilization of MRI as a verification standard for PET becomes unnecessary, thereby enabling researchers to directly compare the efficacy of PET with MRI. Two study included reported similar performance between PET/CT and MRI [16, 20], while three confirmed better performance of PET/CT than MRI [17–19] in PCa detection. It’s worth noting that the performance of PET/CT combined with MRI exhibited significantly higher detection rate of cancerous region comparing to solely MRI [16, 18, 20], indicating that PET may improves the definition of tumor extent. Considering the extensive development and exploration of MRI features [22, 23], PET can serve as an important complement to MRI, which implicates that the integration of PET and MRI have the potential to improve the current clinical guidelines. More details of the above studies are available in Table 1. Further investigations can be conducted to improve PET imaging, with WMH serving as the gold standard for histopathologic validation.
3. *Ultrasound*: Ultrasound (US) has played an important role in prostatic targeted-biopsy, and researchers are now striving to enhance its diagnostic efficacy for PCa. In our included studies, both sector-based registration [24, 25] and complete spatial registration [6] were implemented. With the help of WMH, the combination of Artificial Neuron Network and basic transrectal US could achieve an accuracy of 78% [24]. Besides basic US, Micro-ultrasound (Micro-US) [25] and ultrasound shear wave elastography (USWE) [6] have also shown satisfactory performance with WMH serving as ground truth for validation. These studies indicated the potential for improving US performance with WMH.

#### WMH serve as reference standard in AI radiologist

**Fig. 4:**
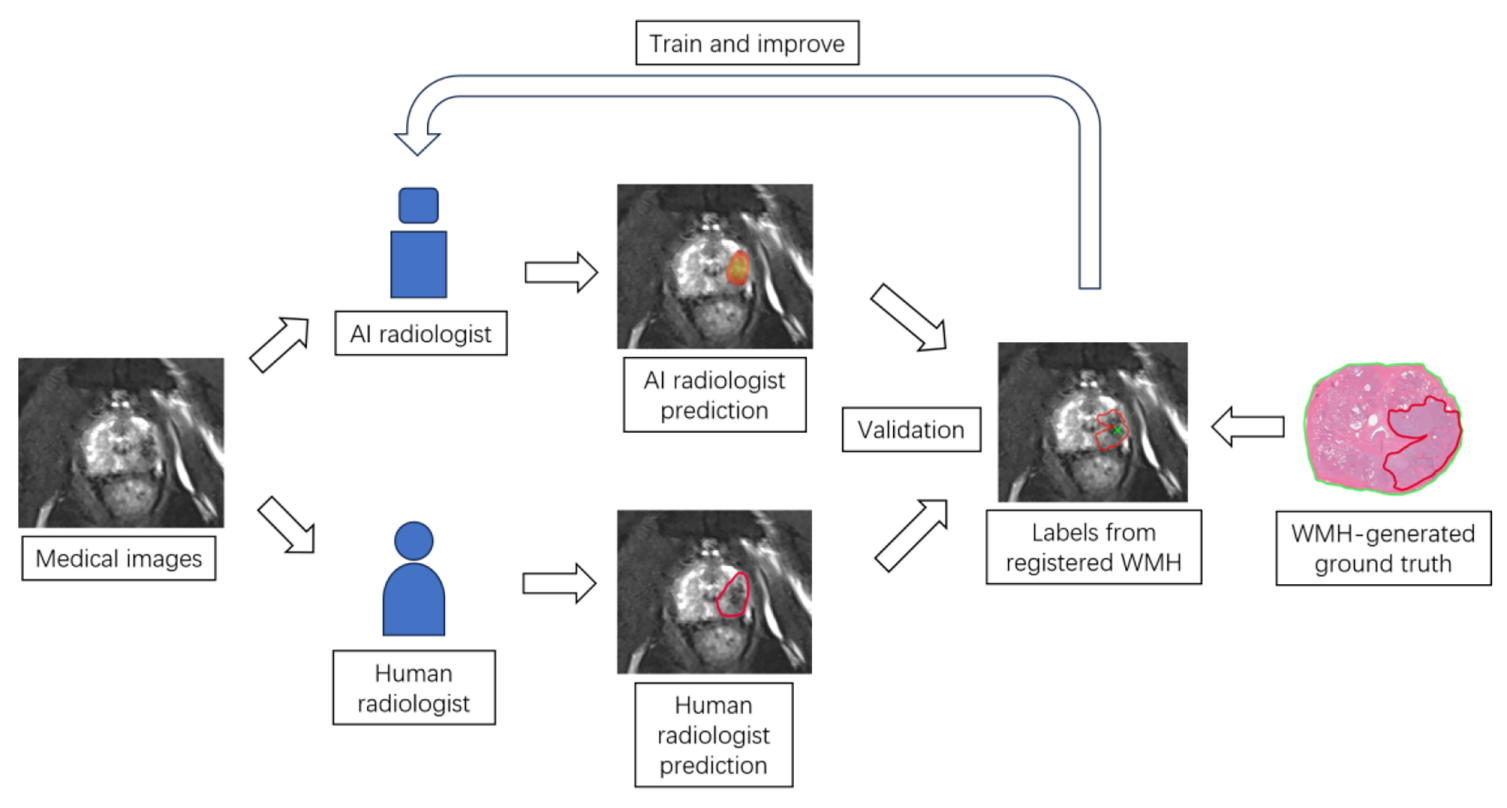
Common protocol of developing AI radiologist using WMH as the ground truth. In the training process, the AI algorithm model will be established by medical images with delineated tumor contours which are labeled using WMH as the ground truth. As the amount of medical images increase, the AI algorithm model will be continuously optimized. Human radiologist labeled tumor contours are used as a control group to evaluate the efficacy of AI algorithm model. After many cycles of iteration, the AI algorithm model will serve as the AI radiologist in future clinical practice. When medical images are received by the AI radiologists, they can generate binary or gradient cancer probability map on the corresponding medical images. AI = artificial intelligence; WMH = whole mount histopathology

Deep learning (DL) is a subfield of artificial intelligence (AI), in which its accuracy gained an evolutional improvement due to the usage of multiple layers of artificial neuron network. WMH is a valuable tool in the development of AI radiologist, serving as the ground truth in both training and validation of AI readers [26–29] **(**Fig. 4**)**. The key advantage of WMH lies in its enhanced legibility and compatibility with medical imaging registration, facilitating the acquisition of registered pairs of medical images and WMH sections for training and validating AI radiologists.

1. *Efficacy of AI radiologists*: The usage of DL largely improved the accuracy of AI radiologist in detection [26, 27] and classification [28, 29] of clinically significant PCa (csPCa). These models have considerably enhanced diagnostic accuracy for both junior and senior radiologists [26]. With the WMH images as ground truth, DL models achieved satisfying classification performance, which reached AUC of 0.75 [28] in detecting csPCa. Notably, one model detected 18% of lesions ignored by radiologists [28], which demonstrated the importance of WMH in establishing AI radiologist models.
2. *Robustness of AI radiologists*: Gunashekar et al. [30] demonstrated that the DL models focus on tumor lesions on WMH ground truth using Class Activation Map. Study by Taimen et al. [31] demonstrated the consistent repeatability of U-Net based DL models in csPCa detection on ADC maps with WMH for validation. Moreover, the ultimate efficacy of DL models is also affected by the labeling strategy employed [32], suggesting that WMH can potentially offer a standardized labeling framework to enhance the robustness of AI radiologists in the future.

### WMH provides comprehensive histopathologic information

WMH preserves the integrity of the prostatic cross-section surfaces, providing comprehensive histopathologic information. It not only facilitates the examination of large tumor lesions and the estimation of the tumor load, but also underscores its substantial value in training AI readers.

#### WMH accelerates tumor volume calculation

**Fig. 5:**
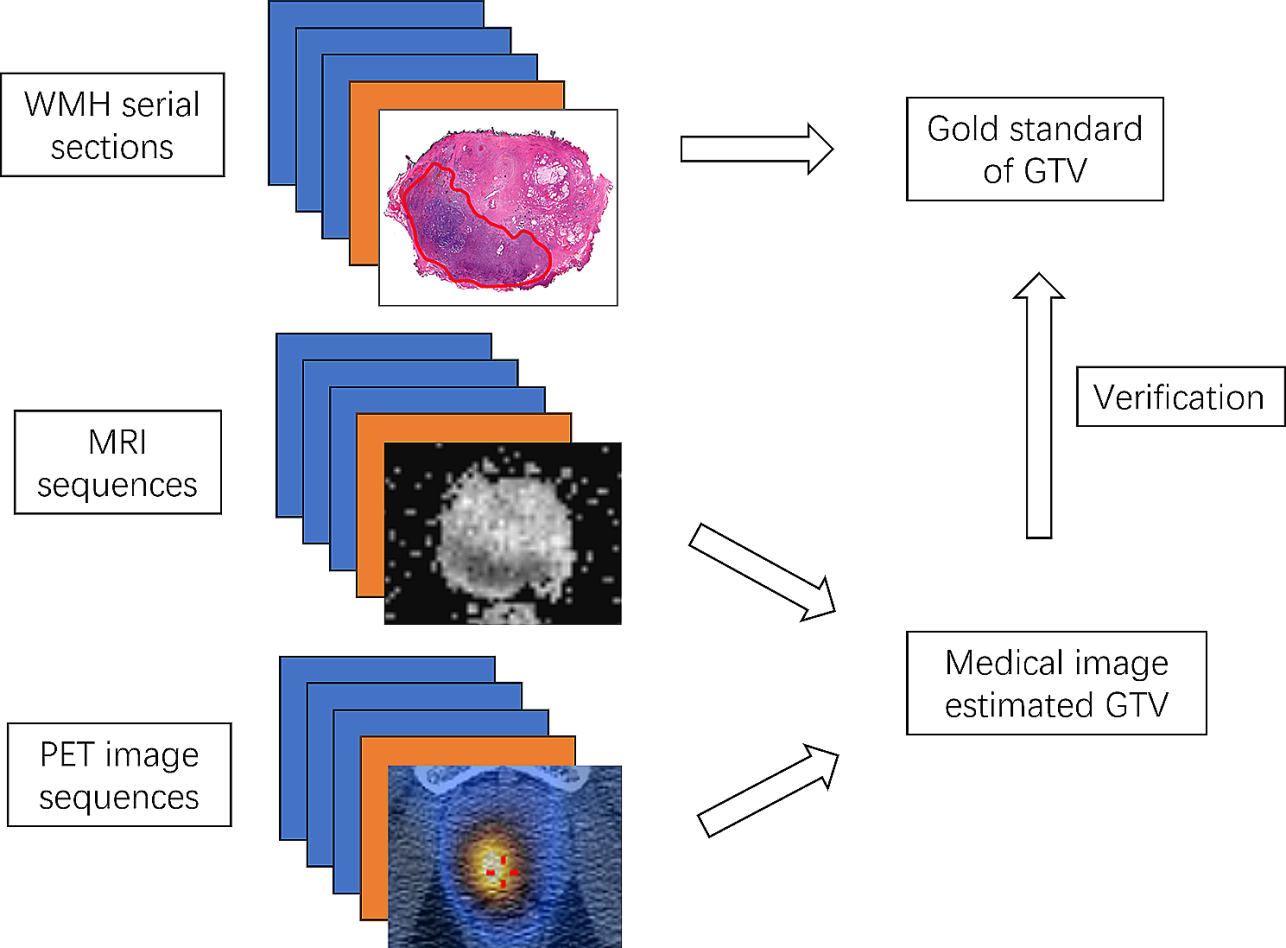
WMH serves as the ground truth in GTV calculation. The GTV is firstly calculated on medical images using tumor area on each slide. Then, the histopathologic tumor volume is calculated on WMH serial section images. Finally, the GTV estimated by medical images is verified with WMH-generated ground truth tumor volume. GTV: gross tumor volume; MRI: magnetic resonance imaging; PET: positron emission tomography; WMH: whole mount histopathology

Gross tumor volume (GTV) estimation involves non-invasively quantifying tumor size using medical imaging methods before initiating treatment. In such studies, the tumor volume obtained from WMH sections serves as the gold standard and is calculated based on the tumor area in each serial section of WMH **(**Fig. 5**)**.

1. *GTV calculation with MRI*: With WMH as reference standard of tumor volume, MRI demonstrated a clear tendency to significantly underestimate the tumor volume [33, 34], especially in cases with smaller size and low Gleason scores [35]. Among all the sequences in MRI, DCE showed best performance in volume estimation tasks, while the underestimation remains significant comparing to WMH-generated ground-truth [34]. Despite the persistent issue of tumor volume underestimation on MRI, Kramer et al. [36] developed a potential solution by using WMH assistance to expand the margin of MRI-delineated tumors by 2 mm, which has shown promising results in improving volume estimation accuracy while avoiding overestimation concerns.
2. *GTV calculation with PET*: In addition to MRI, PET is also a potent technique for GTV estimation for its excellent sensitivity. In a study by Kranz-Rudolph et al. [33], ^68^Ga-PSMA-11-PET/CT significantly outperformed MRI in detection specificity, and overcame the underestimation problem at the same time. Moreover, in a study by Kostyszyn et al. [37], a convolutional neural network (CNN) algorithm was utilized and demonstrated strong agreement in GTV estimation between ^68^Ga-PSMA and ^18^F-PSMA PET images, along with high sensitivities and specificities. With the assistance of WMH, these studies exhibited the superiority of PET in GTV calculation, indicating its substantial value in guiding precise treatment planning in the future.

#### WMH facilitates post-treatment evaluation

In addition to investigating the feature of tumors without medical intervention, can also serve as a valuable tool for evaluating the effectiveness of treatment. The legibility of WMH sections enhances the reading efficacy of pathologists, while the comprehensive information it provides supports the examination of residual PCa and the estimation of tumor burden.

1. *Neoadjuvant therapy*: With WMH providing detailed assessment of pathologic treatment response, Golan et al. [38] evaluated and reported the satisfying outcome in safety and efficacy of lutetium-177 prostate-specific membrane antigen, a radioactive agent in neoadjuvant therapy. WMH can also be employed for investigating the effectiveness of medical imaging in therapeutic evaluation. MRI, for instance, has demonstrated satisfying efficacy in post-treatment residual tumor estimation [39] with the validation of WMH images. Chen et al. [40] also demonstrated a better performance of ^68^Ga-PSMA-11 PET/CT than prostate specific antigen (PSA) response in evaluating pathological response to neoadjuvant therapy using WMH as the reference standard. These studies collectively highlight the potential of WMH in facilitating the assessment following neoadjuvant therapy.
2. *Focal therapy*: Takeda et al. [41] validated the efficacy of the focal therapy inclusion criteria, who retrospective reviewed the WMH sections from all 98 patients meeting the criteria, and extensive PCa was detected in 39 patients, thus highlighting the current limitations of these inclusion. Moreover, Sood et al. [42] developed a novel precision prostatectomy approach, and tested its inclusion criteria through retrospective examination of WMH sections obtained from radical prostatectomy (RP), in which they proved the feasibility of this precision prostatectomy protocol. With the aid of WMH, more reliable treatment planning for focal therapy can be developed, thereby reducing its risk of recurrence and improving prognostic life quality.

#### WMH provides comprehensive histopathologic information for AI readers

WMH sampling method reduces tissue damage to a minimum extent, which is significant for AI readers due to their greater potential than human in identifying subtle features [43]. WMH also offers more legible histopathologic images that are convenient for both management and normalization, thereby largely reduce the difficulty for AI in interpreting histopathologic images.

1. *AI pathologist*: AI pathologists were designed to identify pathologic features directly on histopathologic images. According to a study conducted by Yamamoto et al. [43], a DL-based AI pathologist exhibited superior predictive capabilities compared to human pathologists in recurrence prognosis. This highlights the enormous potential of AI in extracting valuable information from WMH images. Furthermore, studies have found tissue component maps (TCMs) derived from WMH can improve the efficiency of AI pathologists in PCa detection [44] and classification [45]. The above studies indicated that AI pathologist have the potential to identify pathologic features from WMH images automatically in the future, which can facilitate pathological examination and provide gold standard for validation purpose.
2. *AI radiologist*: Other than employing AI pathologists for label generation, studies also involved training AI models with co-registered WMH and MRI. In these studies, AI radiologist was designed to learn from the relationship between WMH and MRI features, thus enabling the identification and correlation of unobservable features on WMH and medical images, which significantly improved the performance of DL models in tumor classification [44, 46]. These studies proved the potential of WMH to serve not only as the ground truth, but also as learning material for AI radiologists.

Overall, the legible and comprehensive histopathologic information provided by WMH can considerably enhance the performance of AI readers, considerably improving the efficacy of human radiologists and pathologists.

### WMH provides comprehensive spatial context

WMH sampling preserves the inherent structure of the prostatic tissue, providing a direct and legible spatial context provided. It facilitates clear visualization of spatial location and distribution patterns of any discernable histopathologic features, resulting in greater convenience for locating cancerous regions or treatment margins.

#### WMH facilitate intraprostatic tumor locating

WMH provides great convenience in locating intraprostatic tumor lesion because the intact tissue was on one physical slide. The spatial context provided by WMH sections offers convenience for the following assignments.

1. *Spatial distribution inspection*: WMH provides a convenient approach to explore the spatial etiology of PCa on prostatic specimens. Research conducted by Zhou et al. [47] discovered that PCa primarily originates from the areas between the apex above 0.5–0.8 cm and the middle slice, and that the inferior half of the prostate had higher positive rate than superior half, offering valuable guidance for the protocol of systematic biopsy and conventional histopathologic sampling procedure.
2. *Feature-location correlation*: Distinguishing the PCa in different regions on WMH, such as peripheral zone (PZ) and transition zone (TZ), can result in more precise diagnosis and more accurate prediction of the tumor feature. In the PZ, ADC has been demonstrated as an effective predictor for PCa, while its efficacy diminishes to varying degrees in the TZ [48]. Other studies on PI-RADS score [49] and MRI-based AI radiologist [29] have found that the diagnosis performance the in PZ was found to be superior to that in the TZ. These findings suggested that the intraprostatic location provided by WMH may offer important implications for clinical PCa detection.
3. *Periurethral PCa locating*: The precise location of periurethral PCa is plays a crucial role in treatment planning for focal therapy. WMH provides legible and comprehensive spatial information of periurethral PCa lesions and urethral lumen, serving as a high-quality histopathologic reference. In two separate studies, MRI method was assessed by the precise location obtained from WMH sections, demonstrating substantial [50] and moderate [51] accuracy, respectively, in identifying periurethral PCa (with distance < 5 mm to urethral lumen). By using WMH as the gold standard, further advancements can be achieved to improve treatment planning for focal therapy.

#### WMH facilitate the identification of extraprostatic tumor lesion

WMH section demonstrates the entire prostatic capsule and its surrounding tissue on a single slide, simplifying the identification of extraprostatic extension (EPE) and seminal vesicle invasion (SVI) and provides valuable insights for treatment decisions and therapeutic planning.

With the validation of WMH-generated ground truth, MRI has shown satisfactory performance in detecting EPE and SVI [52], as well as effectively excluding the occurrence of EPE [53]. In study conducted by Sonni et al. [16], MRI exhibited higher AUC than PET/CT in detecting both EPE (0.79 vs. 0.59, *P* = 0.002) and SVI (0.84 vs. 0.63, *P* = 0.001). These studies utilized WMH as the gold standard for validating medical imaging methods in detecting extraprostatic tumor lesions, which holds the potential for more precise and effective treatment planning.

## Discussion

This article elaborated the advantage of whole mount histopathologic sections in multiple dimensions comparing to conventional sections. Comparing to existing review themed prostate WMH by Cimadamore et al. [2], our review included the integration of WMH with rapid developing imaging methods and AI technology, which were yet not summarized by existing literature. These studies expanded the utilization of WMH for serving as the gold standard in the AI training and validating procedure.

WMH technique exhibited the potential to lead the future in clinical diagnosis and treatment of PCa. The increasing demand for imaging precision necessitates the improvement of existing imaging methods [24, 25] and the exploration of novel imaging technologies [14, 15]. It is foreseeable that WMH will serve as a powerful tool in the innovation and advancement of medical imaging methods. While the human naked eye has limited ability to identify subtle differences [43], artificial intelligence (AI) can extract valuable information at a pixel-wise level, thus have the potential to assist [26], complement [28] or even outperform [43] human in PCa detection, classification, and prognosis prediction. In the foreseeable future, AI can not only complete pre-diagnosis without human intervention [54], thus reduce the diagnosis burden of radiologists, but also integrate clinical features and medical images as input [55, 56], enabling precise and personalized minimally invasive examination and treatment decisions.

With the assistance of the WMH technique, further research should be conducted to explore the immense potential of AI.

There are limitations utilizing WMH in clinical research. While retrospective studies contribute significantly to clinical research evidence, they are inherently limited by their susceptibility to various forms of bias. This is particularly true when considering patients who have access to WMH examinations, as they must first undergo RP, which will possibly cause selection bias and may affect the generalizability of medical imaging protocols. These facts indicated that more prospective studies are required across diverse fields to generate more convincing evidence utilizing WMH. Moreover, the production of WMH sections requires special consumables and machines, and the proficiency in analyzing WMH sections necessitates pathologists to gain experience from substantial training for its special size comparing with conventional histopathology.

Our review has some limitations. Given the variety of imaging techniques, we focused on the most used techniques, MRI, PET, and US. By including articles published after 2019, we can effectively capture the studies in the AI rapid developing area. These methods might lead to selection bias. It should also be noted that some articles may have utilized the WMH technique without explicitly mentioning it in the title or abstract, potentially leading to their exclusion from our search. These factors may impact the overall comprehensiveness of our overview of current studies.

## Conclusions

The utilization of whole mount histopathology (WMH) in the clinical diagnosis and treatment of prostate cancer (PCa) offers unparalleled advantages. WMH exhibits enhanced legibility, which improves the efficacy of pathologic examination of prostate cancer and provide educational value for pathologists training. As a gold standard, WMH provide comprehensive histopathology information as well as spatial information. Therefore, it acts like bridges for connecting molecular imaging, ultrasound detection, and functional MRI imaging, with the helping of artificial intelligence. WMH based pre-trained models will change the rules in US guided prostate biopsy and focal therapy, also provide better outcomes for prostate cancer patients.

## Data Availability

All data generated or analyzed during this study are included in this published article.

## Abbreviations

ADC: apparent diffusion coefficient
AI: artificial intelligence
AUC: area under the curve
CT: computed tomography
DCE: dynamic contrast enhanced
EPE: extraprostatic extension
GS: Gleason grade
mpMRI: multiparametric magnetic resonance imaging
MRI: magnetic resonance imaging
PCa: prostate cancer
PET: positron emission tomography
PI-RADS: Prostate Imaging Reporting and Data System
PSMA: prostate-specific membrane antigen
PZ: peripheral zone
SUV: standardized uptake value
SVI: seminal vesicle invasion
T: Tesla
TSC: tissue sodium concentration
TTP: time-to-peak
TZ: transition zone
US: ultrasound
